# An Exploratory Study to Determine Whether *BRCA1* and *BRCA2* Mutation Carriers Have Higher Risk of Cardiac Toxicity

**DOI:** 10.3390/genes8020059

**Published:** 2017-02-02

**Authors:** Monique Sajjad, Michael Fradley, Weihong Sun, Jongphil Kim, Xiuhua Zhao, Tuya Pal, Roohi Ismail-Khan

**Affiliations:** 1 H. Lee Moffitt Cancer Center and Research Institute, Tampa, FL 33612, USA; moniquesajjad@gmail.com (M.S.); Weihong.sun@moffitt.org (W.S.); jongphil.kim@moffitt.org (J.K.); xiuhua._zh@yahoo.com.org (X.Z.); tuya.pal@moffitt.org (T.P.); 2 Division of Cardiovascular Medicine, University of South Florida, Tampa, FL 33620, USA; Mfradley@health.usf.edu

**Keywords:** anthracycline-related cardiac toxicity, *BRCA1* mutation, *BRCA2* mutation, conduction abnormalities, heart failure

## Abstract

Anthracycline-based cardiotoxicity is concerning for women with breast cancer and portends a dose-dependent risk of developing left ventricular dysfunction. Overall, the prevalence of heart failure (HF) is ≈2% of the total US population; however, *BRCA*-deficient mice have shown increased HF. We evaluated for the inherent risk of HF in women with *BRCA* mutations to determine whether treatment with anthracycline-based therapy increased this risk. We obtained results on *BRCA* mutation carriers regarding cancer treatment and HF, identified through the *BRCA* patient advocacy organization Facing Our Risk for Cancer Empowered (FORCE) and the Moffitt-based Inherited Cancer Registry. In our patient group (232 *BRCA1* and 159 *BRCA2* patients; 10 with both mutations), 7.7% reported HF, with similar proportions in *BRCA1* versus *BRCA2* carriers (7.4% and 8.2%, respectively). These proportions are significantly higher than published rates (*p* < 0.001). There was no statistically significant difference in HF rates comparing anthracycline-treated versus anthracycline-naïve patients however (7.1% vs. 8.3%; *p* = 0.67). In addition, 9.1% of *BRCA1* carriers and 8.2% of *BRCA2* carriers reported arrhythmias. *BRCA* mutation carriers showed increased risk of cardiotoxicity versus the general population and an overall increased risk of cardiotoxicity from anthracycline-based therapy. Our study supports data that *BRCA* carriers have increased non-cancer mortality from cardiotoxicity. A prospective trial to determine HF and conduction abnormalities in this population is warranted.

## 1. Introduction

Overall, approximately 5%–10% of breast and ovarian cancer cases are due to mutations in the high-penetrance genes *BRCA1* and *BRCA2* (*BRCA*) [1]. It is well-established that patients with mutations in the *BRCA* genes have increased mortality due to malignancy [2]; however, a recent study has also suggested a significant association between *BRCA* mutations and increased non-cancer mortality (*p* = 0.024) [3]. Mutation carriers have a 56%–87% risk of developing breast cancer by the age of 70 [2,4]. Lifetime risks for ovarian cancer are up to 44% and 27% for *BRCA1* and *BRCA2*, respectively [1].

Prevalence of heart failure (HF) in the general population of American women varies and increases with age. According to American Heart Association data, this risk varies between 0.3% in women under 30 and 11.8% in the elderly [5]. The overall risk for the general US population including males and females is estimated at 2% [5]. It is well-established that anthracycline use can lead to heart failure. The cardiotoxic effects of anthracycline therapy are seen in a dose-dependent fashion. The risk of heart failure due to adriamycin at 450 mg/m^2^ is 3%–4% [6].

This exploratory study was performed to determine whether patients with *BRCA* mutations are at a higher inherent predisposition for cardiac disease. We also sought to determine whether mutation carriers were at a higher risk for toxicity from anthracycline therapy, which is one of the current standards of care for chemotherapy in this population. Third, we attempted to identify other possible causes responsible for increased mortality in these patients. 

## 2. Materials and Methods 

This study received Institutional Review Board approval (number Pro00004774; 20/06/2014). Patients with *BRCA1* or *BRCA2* mutation status were offered enrollment in the patient advocacy groups known as Facing Our Risk for Cancer Empowered (FORCE) and Inherited Cancer Registry (ICARE). FORCE is a nonprofit organization devoted to patients and family members with *BRCA* mutations. This organization meets annually and has over 13,000 members nationwide. We also identified patients using the ICARE initiative at the Moffitt Cancer Center (see Appendix A). Through FORCE and ICARE, the surveys were available to over 13,000 patients. Eligible patients included females with confirmed *BRCA1* or *BRCA2* mutations of any age and any treatment history. Patients were invited to participate in an optional online or paper survey (see Supplementary Survey S1) regarding general adverse effects surrounding their breast cancer treatment and overall health. Patients were consented by voluntarily completing the survey and had the option to give additional consent to be contacted for follow-up questions. Four hundred one surveys were completed, and all were included for analysis. The survey consisted of 57 questions, including a thorough patient-reported review of the cardiovascular system and disease states. Specific information regarding baseline cardiac risk factors and medical history prior to therapy was also obtained. Age, prior or current tobacco history, diabetic status, cholesterol levels, and history of cardiac disease were assessed. Targeted questions included Number 8 (cardiac)—“Did you experience any of the following: irregular rhythm, prior heart attack, heart failure”—Number 25—“Was your heart function normal before starting treatment?”—Number 26—“Is your heart function normal now?”—Number 42b—“Have you been diagnosed with heart attack?”—and Number 42c—“Have you been diagnosed with heart failure?” The survey also included detailed questions to determine preexisting conditions and comorbidities. Data of patients who underwent treatment at Moffitt Cancer Center were reviewed. Attempts were made to contact patients treated at outside centers, with questionnaire responses suggestive of cardiovascular complications.

The next part of the study included assessing the cardiac risk profiles of patients who had specifically been treated with anthracycline chemotherapy, comparing these values to established historical data. We contacted patients who answered “Yes” to any of the targeted questions (see above) and gave permission to do so. Of the 31 patients with heart failure, only 8 agreed to be contacted. They were all able to provide proof of HF diagnosis via clinical history or echo report. The survey, including targeted cardiac questions, is included in Supplementary Survey S1.

Population characteristics were summarized using descriptive statistics: frequency and proportion for categorical variables and median and range for continuous variables. The exact binomial distribution was used to compute the 95% confidence interval (CI) for proportions and to compare those with the proportions of a well-established population control. The association between two categorical variables was evaluated by the chi-square test.

## 3. Results

### 3.1. Population Characteristics

**Figure 1:**
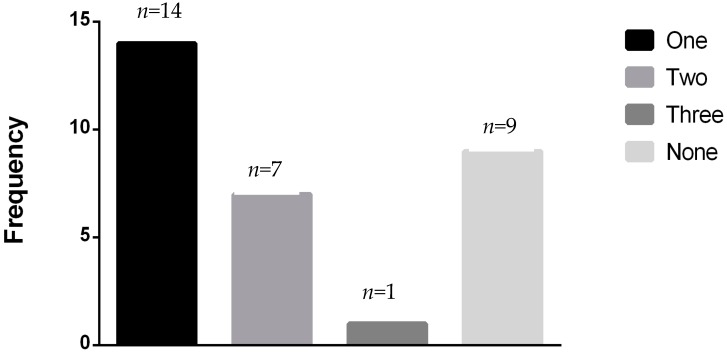
Number of confounding risk factors seen in the 31 *BRCA* patients with heart failure.

A total of 401 patients were entered into the study: 232 were *BRCA1* carriers, and 159 were *BRCA2* carriers (see Table 1 for patient characteristics). Ten patients carried both mutations. Patient accrual was initiated in April 2010 and concluded in November 2010 after 400 surveys were completed. All patients were female. Age ranged from 40 to 76 years. The patient cohort was generally healthy, and most patients with heart failure had 0–1 risk factor (see Figure 1).

Thirty-one patients (7.3%) were diagnosed clinically with HF. Of these, 17 patients (7.3%) carried *BRCA1* mutations and 13 patients (8.2%) carried *BRCA2* mutations. One patient that carried both mutations was diagnosed with heart failure (Table 2).

When compared with rates of heart failure in the overall population, this proportion in our patient population was significantly higher (7.7% vs. 2%; *p* < 0.001). When adjusted for age, *BRCA* carriers in the 40- to 59-year-old age group (15/264 patients) had a 5.7% risk (CI, 3.2–9.2) versus 0.8% in the general population of women (*p* < 0.0001), and patients in the 60- to 79-year-old age group (12/76 patients) had a 15.8% risk (CI, 8.4–26.0) versus 5.4% in the general population (*p* < 0.0001). Four patients could not be included in age stratification because they did not include their age on the survey.

There was no difference between risk in *BRCA1* and *BRCA2* carriers who had 7.3% and 8.2% incidence of heart failure, respectively (*p* = 0.76). Of the 7.7% of *BRCA1* and *BRCA2* mutation carriers who developed heart failure, 8.3% were anthracycline naïve (statistically significant at *p* ≤ 0.001) compared with the general population risk of 2%. This indicates that *BRCA* carriers may have a higher inherent risk of heart failure than the general population even when anthracycline use is excluded. In our patient group, 7.1% of mutation carriers developed heart failure after receiving anthracycline therapy with either adriamycin or epirubicin. When compared to the known risk of heart failure from anthracycline therapy of 3%, *BRCA* mutation carriers had a higher risk of cardiotoxicity from anthracycline therapy (*p* = 0.008) (Table 3). There was no statistically significant difference in the incidence of HF between the anthracycline-treated patients and those who were chemotherapy naïve (7.1% vs 8.3%; *p* = 0.67).

In addition, 21 of 232 (9.1%) *BRCA1* carriers and 13 of 159 (8.2%) of *BRCA2* carriers reported arrhythmias. Overall, 37 patients of 391 (9.2%) reported arrhythmias (Table 4). 

## 4. Discussion

There is clear evidence of increased non-malignant mortality in *BRCA* patients [3]. In our study, we sought to determine whether this risk was related to cardiac mortality, namely, heart failure. A recent study of 81 patients showed no change in ejection fraction, but this may be due to a small sample size and a short duration of follow-up [7].

*BRCA1* is a human tumor suppressor gene that produces the breast cancer type 1 susceptibility protein. *BRCA1* is expressed in cells of the breast and other tissues, where it functions to repair or destroy damaged DNA and protect cells against oxidative and genotoxic stress. Mutations in this gene alter function and allow for tumors to arise [8]. Thus, mutations in this gene could lead to pathologic pathways, predisposing to physiologic dysfunction caused by oxidative stress, particularly cardiovascular disease. In support of this theory, murine studies conducted with cardiac myocytes revealed that wild-type *BRCA1* is essential to limiting apoptosis and improving cardiac function in response to genotoxic stress (doxorubicin) and oxidative stress (ischemia) [9]. Furthermore, heart-specific *BRCA1* deletion has been shown to promote severe systolic dysfunction and limited survival in mice [9]. *BRCA2* deletion has led to increased cardiomyocyte apoptosis after anthracycline therapy [10]. Follow-up studies reported an association between single nucleotide polymorphisms in *BRCA1*-associated protein and myocardial infarction risk in a large Japanese patient cohort with replication in additional Japanese and Taiwanese patient cohorts [11]. Even in women who do not receive anthracycline-based chemotherapy, BRCA protein deficiency can increase their susceptibility to any form of oxidative stress, whether it is from other types of cancer treatments, early surgical menopause, subclinical ischemia, or endothelial dysfunction from hypertension, hyperlipidemia, diabetes, or insulin resistance (which is known to be increased in the setting of BRCA protein deficiency) [12,13,14]. These mechanisms could play a role in the inherently higher risk for heart failure reported in those patients exposed to anthracyclines and those who were chemotherapy naïve. 

Our study participants reported a significantly increased risk of heart failure after anthracycline therapy compared with the general population. The cardiotoxic effects of anthracyclines have been well-studied in cancer patients but have not been directly correlated to patients with *BRCA* mutations. Hypothesized mechanisms of anthracycline-induced cardiotoxicity include free radical damage to myocytes, which can occur non-enzymatically through direct interaction with iron [1] or enzymatically through interaction with cardiolipin. Anthracyclines act as reducing agents generating free radicals in both pathways and are shown to disrupt DNA synthesis by interference with topoisomerase, leading to apoptosis [1]. Because *BRCA1* is associated with DNA repair, mutations may portend a higher risk of damage by anthracycline therapy.

Our secondary goal of this study was to identify other possible mechanisms to explain the increased non-cancer-related mortality in this population. By means of the encompassing survey, we also found an alarming number of otherwise healthy patients who reported arrhythmias. There are no well-established statistics for the overall rates of arrhythmias in women in the United States; therefore, our question regarding arrhythmias should have been more specific, so that more detailed statistical analysis could have been performed. However, the most common conduction abnormalities are first-degree atrio-ventricular blocks, with a 3% risk in black women and a 1.3% risk in white women, and atrial fibrillation, which ranges from 6.6 per 100,000 women per year for 15- to 44-year-olds and 1203.7 per 100,000 women per year for those ≥85 years of age [15,16]. These rates are much lower than the percentages that we found in our study. Because our survey was not initially formatted with detailed questions regarding arrhythmias, we did not have data as to specific types, durations, and treatments required for the arrhythmias found in our patient cohort.

The major limitation of this exploratory study was the inability to contact all participants for further information that was not listed in the primary survey. This was an online survey reaching patients across multiple institutions. Because this was an anonymous survey, we were unable to further discuss specific survey responses and obtain objective data from a large percentage of participants, unless they provided consent and contact information for further communication. The data collected also consisted of patients’ self-reporting their diagnoses and was limited to the questions addressed in the survey. Moreover, objective confirmatory data were unavailable in most cases. Of the contacted patients who reported heart failure, echocardiogram reports did confirm low ejection fraction when available. However, we considered the diagnosis valid since heart failure is a clinical diagnosis and the targeted questions were specific. Among the patients who reported arrhythmias, electrocardiograms confirmed the lack of sinus rhythm when available. An additional weakness was that these data were compared with historical controls, allowing different biases to be introduced, including selection bias given the inherent differences in the populations compared. Finally, the use of trastuzumab was not addressed in this study. However, the use of this therapy is less common in BRCA breast cancer patients, as most have triple negative tumors [17]. This may need to be addressed in the future if these data are confirmed with prospective studies.

## 5. Conclusions

Prior studies in humans have sought to determine why *BRCA* carriers have increased non-cancer mortality. Previous studies have indicated that this may be due to cardiotoxicity. The findings of our cross-sectional study design suggest this as well. We consider this an interesting hypothesis-generating observation; however, it remains important to conduct larger studies to test this hypothesis, ideally with prospective follow-up and objective record verification. A more in-depth review of cardiovascular effects in this patient population is warranted and currently underway. The prospective study will compare ejection fraction in *BRCA* patients with wild-type counterparts before and after treatment with anthracycline.

## Figures and Tables

**Table 1:** Patient characteristics.

	Number of Patients (%) (*n* = 401)
*BRCA* status *BRCA1* *BRCA2* *BRCA1* and *BRCA2*	232 (57.9) 159 (39.7) 10 (2.5)
Hypertension	97 (24.2)
Diabetes	18 (4.5)
Hyperlipidemia	207 (51.6)
Tobacco use	37 (9.2)
Age group 20–39 years 40–59 years 60–79 years No answer	28 (7.0) 264 (65.8) 76 (19.0) 33 (8.2)

**Table 2:** Patients diagnosed with heart failure.

Patient Group	Heart Failure, No. of Patients (%)	No Heart Failure	Total	95% Confidence Interval (%)	*p* Value Compared to 2%
*BRCA1*	17 (7.3%)	215	232	4.3–11.5	<0.0001
*BRCA2*	13 (8.2%)	146	159	4.4–13.6	<0.0001
*BRCA1* and *BRCA2*	1 (10%)	9	10	0.3–44.5	0.37
Total	31 (7.7%)	370	401	5.3–10.8	<0.0001

**Table 3:** Heart failure risk in BRCA patients with anthracyline therapy.

	Number of Patients				% HF on Anthracycline	% HF Chemotherapy Naïve
	Received Anthracycline	Chemotherapy Naïve	HF on Anthracycline	HF Chemotherapy Naïve		
*BRCA1*	106	126	6	11	5.7	8.7
*BRCA2*	70	89	6	7	8.6	7.9
Both	7	3	1	0	14.2	0
Total	183	218	13	18	7.1	8.3

Abbreviations: HF, heart failure.

**Table 4:** Arrhythmias in BRCA patients.

Patient Group	Irregular Rhythm, *n*	No Irregular Rhythm or Unknown, *n*	Total, *n*	Irregular Rhythm, % (95% CI)
*BRCA1*	21	211	232	9.1 (5.7–13.5)
*BRCA2*	13	146	159	8.2 (4.4–13.6)
*BRCA1* and *BRCA2*	3	7	10	30.0 (6.7–65.3)
Total	37	364	401	9.2 (6.6–12.5)

## Supplementary Materials

The following are available online at http://www.mdpi.com/2073-4425/8/2/59/s1, S1: Cancer therapy and side effects in BRCA patients.

## Appendix A

**ICARE:** The ICARE initiative, based at Moffitt, is an inherited cancer registry and database (grant funded) and initiated by Dr. Tuya Pal. Christina Bittner, a Board-Certified Genetic Counselor (a member of the ICARE team), contacts patients with resources that may be of interest to the BRCA patient.

**FORCE:** The FORCE organization was established in 1999 and offers expert-reviewed research information, resources, advocacy, and peer support for individuals and families affected by hereditary cancers. FORCE efforts include an annual conference, print and electronic newsletters, a comprehensive website, a toll-free helpline, webinars and webcasts, regional support groups, educational brochures, and chat rooms through which individuals can access the latest information on treatment, risk-management, prevention, psychosocial and quality-of-life issues, and research studies, as well as discuss and share the cancer-related issues that they are facing.

FORCE is the only international, nonprofit organization devoted to improving the lives of individuals and families affected by hereditary breast and ovarian cancer. Their mission includes providing up-to-date information, resources, peer support, and advocacy, raising awareness, and promoting research specific to hereditary cancer. FORCE maintains a print newsletter mailing list of about 13,000 individuals and an e-mailing list of approximately 10,000 individuals, growing at a rate of approximately 100 contacts monthly. FORCE has collaborations with major cancer centers and cancer advocacy groups. FORCE is a partner member of the Ovarian Cancer National Alliance (OCNA) and the Allied Support Group for Ovarian Cancer (organized by SGO).
