# Supplementary material for: An Exploratory Study to Determine Whether BRCA1 and BRCA2 Mutation Carriers Have Higher Risk of Cardiac Toxicity
Source: Genes (Basel). 2017 Feb 2;8(2):59. doi: 10.3390/genes8020059 (PMC5333048; doi:10.3390/genes8020059)
Supplement: Supplementary file 1 [file genes-08-00059-s001.pdf]

Study ID:Pro00004774 Date Approved: 6/20/2011 Expiration Date: 6/20/2012

Cancer therapy and side effects in BRCA patients

H. Lee Moffitt Cancer Center/ FORCE

Study ID #: | | | | |

Patient ID #: | | | | | | |

For questions or comments, please contact:

Roohi Ismail-Khan, MD
Department of Women's Oncology
H. Lee Moffitt Cancer Center
12902 Magnolia Drive
Tampa, FL 33612, USA
Telephone #: 813-745-4933
Email Address: roohi.ismail-khan@moffitt.org

USF
UNIVERSITY OF SOUTH FLORIDA

Moffitt Cancer Center / University of South Florida (USF)

Informed Consent to Participate in Research
Information to Consider Before Taking Part in this Research Study

You are being asked to take part in a research study. Please read this information carefully and take your time making your decision.

We are asking you to take part in a research study called: **Cancer therapy and side effects in BRCA patients.**

The person who is in charge of this research study is **Roohi Ismail-Khan, MD**. This person is called the **Principal Investigator**. However, other research staff may be involved and can act on behalf of the person in charge.

The research will be conducted at **Moffitt Cancer Center.**

The purpose of this study is to determine if BRCA carriers are at a higher risk for cardiac diseases compared to people without BRCA.

If you decide to take part in this study, you will be asked to complete the following survey. The survey asks questions about your medical history, specifically if you have had cancer or heart disease and if you have any other risk factors for these diseases. If there are questions you do not feel comfortable answering, you do not have to answer them.

You have the option of completing the survey anonymously, without providing any personal information that could identify you. Only the information you provide in the survey will be used in the research. At the end of the survey you are also given the option of providing the research team your contact information so that they can reach you for further information. Our goal is to have 400 people complete the survey.

You do not have to participate in this research study. Completing the survey is completely optional, as is providing information to be contacted later.

You will not personally benefit from being in this study, but what we learn from this study may help people with the BRCA1 mutation in the future.

This research is considered to be minimal risk. That means that the risks associated with this study are the same as what you face every day. There are no known additional risks to those who take part in this study.

Moffitt Cancer Center Injury Statement

In the event that you sustain an injury as a result of participating in this research, monetary compensation for damages is not automatically available. Damages are only available to the extent specified in Florida law (Florida Statute 768.28). This statue provides that damages are available only to the extent that negligent conduct of an employee caused your injuries and are limited by law. If you believe you are injured as a result of participation in this research and the negligent conduct from one of the staff conducting the study, you may notify the Risk Manager at (813) 745-4219.

You do not give up any of your legal rights by agreeing to participate.

We will not pay you for the time you volunteer while being in this study.

There is no cost to take part in this survey.

Authorization to Use and Disclose Protected Health Information

In this research study, we use and share your health information to the extent authorized (permitted) by you. We know that this information is private. The federal privacy regulations of the Health Insurance Portability & Accountability Act (HIPAA) protect your identifiable health information. If you authorize us to use your information we will protect it as required by the law.

Research at Moffitt Cancer Center is conducted jointly with the University of South Florida. By signing this form, you are permitting Moffitt Cancer Center and the University of South Florida to use personal health information collected about you for research purposes. You are also allowing Moffitt Cancer Center to share your personal health information with individuals or organizations other than Moffitt Cancer Center and USF who are also involved in the research and listed below.

We may publish what we find out from this study. If we do, we will not let anyone know your name. We will not publish anything else that would let people know who you are.

Federal law says we must keep your study records private. Unless you give us your name, the results of your survey will not contain information that would identify you. We will keep the records of this study private by keeping them in a locked area of the Breast Cancer Department at Moffitt Cancer Center. The following people and/or organization(s) will be allowed to disclose, use, and receive your information for the research purposes set forth in this form, but they may only use and disclose the information to the other parties on this list, to you or your personal representative, or as permitted by law:

Every research site for this study, including the Moffitt Cancer Center and University of South Florida, and each site's study team, research staff and medical staff;

Every member of the Moffitt Cancer Center and University of South Florida workforce who provides services in connection with this study;

Any federal, state or local governmental agency that regulates the study (such as the Food and Drug Administration (FDA) and Florida Department of Health (FDH);

The designated Protocol Review and Monitoring Committees, Institutional Review Boards, Privacy Boards, Data and Safety Monitoring Board and their related staff that have oversight responsibilities for this study;

The National Cancer Institute in evaluating the ongoing research of the Moffitt Cancer Center as a Comprehensive Cancer Center;

The organizations and people listed above may employ or pay various consultants and companies to help them understand, analyze and conduct this study. All of these people may not be known now, but if you would like to have more specific information about this at any time during the study, you may ask the study doctor and your questions will be answered.

Moffitt Cancer Center and the University of South Florida cannot guarantee the privacy of your information, or block further use or distribution, after the information has left MCC or USF. The sponsor of this study or others listed above may further disclose your information. If disclosed, the information may no longer be covered by federal privacy regulations.

Anyone listed above may use consultants in this research and for the purpose of this study, may share your information with them. Individuals who receive your health information for this research study may not be required by the HIPAA Privacy Rule to protect it and may share your information with others without your permission. They can only do so if permitted by the laws governing them.

By completing the survey, you authorize the use and disclosure of the information you provide in the survey. The purpose for the uses and disclosures you are authorizing is to conduct the study explained to you above and to ensure that the information relating to that study is available to all parties who may need it for research purposes.

USF UNIVERSITY OF SOUTH FLORIDA

By completing the survey, you authorize the use and/or disclosure of your protected health information described above. Your authorization to use your health information will never expire unless and until you expressly revoke it in writing to:

Principal Investigator's name: Roohi Ismail-Khan, MD

Address of the PI: Moffitt Cancer Center
12902 Magnolia Drive, Mail Stop: MCC-BR Prog
Tampa, FL 33612-9497

You should only take part in this study if you want to volunteer. You should not feel that there is any pressure to complete the survey. There will be no penalty or loss of benefits you are entitled to receive if you do not complete the survey.

If you have any questions, concerns or complaints about this study, or experience an adverse event or unanticipated problem, call Dr. Roohi Ismail-Khan at 813-745-4933.

If you have questions about your rights as a research patient at Moffitt Cancer Center, call the Division of Research Compliance in the Corporate Compliance Department at The Moffitt Cancer Center at (813) 745-1869.

If you have questions about your rights as a participant in this study, general questions, or have complaints, concerns or issues you want to discuss with someone outside the research, call the USF IRB at (813) 974-5638.
By completing the survey you are giving your consent to participate in this research study and authorization to use your anonymous information.

If you do not want to participate, **STOP HERE**

STUDY ID: ☐☐☐☐ PATIENT ID: ☐☐☐☐☐☐

This is a survey to determine side effect risks for patients with BRCA mutations compared to the general population. By participating in and completing this survey, you are giving permisssion to the study coordinator and staff to use the information you provide anonymously for research and publication purposes. You will not be identified in any studies or publications related to this survey. *(Please use a blue or black ink pen to complete the survey)*.

Are you a Moffitt patient? ☐ Yes ☐ No

1. Are you Female? ☐ Yes ☐ No

2. Date of Birth? **(Optional)** ☐☐ / ☐☐ / ☐☐☐☐ (mm / dd / yyyy)

General Cancer History in BRCA

3. Have you ever tested positive for the BRCA-1 gene mutation?
   ☐ Yes ☐ No ☐ I don't know, but I have a strong family history of cancer

4. Have you ever tested positive for the BRCA-2 gene mutation?
   ☐ Yes ☐ No ☐ I don't know but I have a strong family history of cancer

5. Have you ever been diagnosed with cancer? ☐ Yes ☐ No
   5a. Was it breast? ☐ Yes ☐ No
   If yes,
   Was it lobular? ☐ Yes ☐ No
   Was it ductal? ☐ Yes ☐ No
   Was it another type? ☐ Yes ☐ No
   If so, please specify: ____________________
   5b.Was it ovarian? ☐ Yes ☐ No
   5c.Was it other? ☐ Yes ☐ No
   If so, please specify: ____________________

6. At diagnosis, what stage were you?
   ☐ Stage I ☐ Stage II ☐ Stage III ☐ Stage IV

7. When were you first diagnosed? ☐☐ / ☐☐☐☐ (mm / yyyy)

Review of Systems

8. Did you experience any of the following side effects:

	Yes	No	Don't know
**General:**			
Weight loss	☐	☐	☐
Weight gain	☐	☐	☐
Fatigue	☐	☐	☐
Fever	☐	☐	☐
Night sweats	☐	☐	☐
Loss of appetite	☐	☐	☐

USF
UNIVERSITY OF SOUTH FLORIDA

STUDY ID: PATIENT ID:

	Yes	No	Don't know
**Skin:**			
Rash	☐	☐	☐
Itching	☐	☐	☐
Hair Loss	☐	☐	☐
Nail changes	☐	☐	☐
**Breast:**			
Breast tenderness	☐	☐	☐
Breast swelling	☐	☐	☐
Nipple discharge	☐	☐	☐
**HEENT:**			
Headaches	☐	☐	☐
Vertigo	☐	☐	☐
Lightheadedness	☐	☐	☐
Vision changes	☐	☐	☐
Nose bleeding/drainage	☐	☐	☐
Frequent colds	☐	☐	☐
Dental difficulties	☐	☐	☐
Mouth pain	☐	☐	☐
Trouble swallowing	☐	☐	☐
**Cardiac:**			
Chest pain	☐	☐	☐
Irregular rhythm	☐	☐	☐
Palpitations/racing heart	☐	☐	☐
Trouble breathing/shortness of breath?	☐	☐	☐
If so, was it worse with exertion?	☐	☐	☐
Leg swelling	☐	☐	☐
Prior heart attack	☐	☐	☐
Heart failure	☐	☐	☐
History of high blood pressure	☐	☐	☐
History of heart surgery	☐	☐	☐
**Lungs:**			
Wheezing	☐	☐	☐
Cough	☐	☐	☐
Any sputum?	☐	☐	☐
Any blood?	☐	☐	☐
**GI:**			
Abdominal pain	☐	☐	☐
Heartburn	☐	☐	☐
Nausea	☐	☐	☐
Vomiting	☐	☐	☐
Constipation	☐	☐	☐
Diarrhea	☐	☐	☐

STUDY ID: ☐☐☐☐ PATIENT ID: ☐☐☐☐☐☐

	Yes	No	Don't know
**GU:**			
Trouble urinating	☐	☐	☐
Loss of libido	☐	☐	☐
Change in menstruation	☐	☐	☐
**Musculoskeletal:**			
Pain in muscles	☐	☐	☐
Arthritis/ joint pain	☐	☐	☐
**Neuro:**			
Stroke	☐	☐	☐
Weakness in extremities	☐	☐	☐
Tremor	☐	☐	☐
Numbness or tingling in extremities	☐	☐	☐
**Psych:**			
Anxiety	☐	☐	☐
Depression	☐	☐	☐
**Hematologic:**			
Anemia	☐	☐	☐
Bleeding tendency	☐	☐	☐
Easy bruising	☐	☐	☐

GYN (FEMALES ONLY)

9. What age did you start having periods? ☐☐

10. Are you:
- ☐ Pre-menopausal (I still have regular menses)
- ☐ Peri-menopausal (I am going through the process)
- ☐ Post-menopausal (My menses have stopped)
- ☐ Surgical menopausal (both ovaries were removed by surgery)
- ☐ Status post hysterectomy (ovaries have not been removed but uterus was removed)
- ☐ Other

10a. What age did you start experiencing menopause? ☐☐

11. Were you ever on oral birth control? ☐ Yes ☐ No

11a. If yes, how many years? ☐☐

12. Were you ever on hormonal therapy such as estrogen or progesterone replacements? ☐ Yes ☐ No

12a. If yes,

Was it pills? ☐ Yes ☐ No

Was it cream? ☐ Yes ☐ No

STUDY ID: ☐☐☐☐ PATIENT ID: ☐☐☐☐☐☐

13. How many times have you been pregnant? ☐☐

14. How many live births have you had? ☐☐

Social History

15. Did you smoke cigarettes at the time of diagnosis? ☐ Yes ☐ No

16. Do you smoke now? ☐ Yes ☐ No

17. Do you/did you drink alcohol? ☐ Yes ☐ No

17a. If yes, how often:

☐ less than 5 drinks per week ☐ 5-10 drinks per week ☐ 10-15 drinks per week ☐ more than 15 drinks per week

18. How much did you weigh at the time of diagnosis? ________ lbs

19. How much do you weigh now? ________ lbs

20. How tall are you? _____ ft _____ inches

21. How often do you exercise?

☐ less than 3 times per week ☐ 3-5 times per week ☐ more than 5 times per week

22. Do you work ? ☐ Part time ☐ Full time ☐ Unemployed ☐ Retired

23. Are you disabled? ☐ Yes ☐ No

Cardiac Risk Factors/ Cardiac History

25. Was your heart function normal before starting treatment? ☐ Yes ☐ No ☐ I don't know

26. Is your heart function normal now? ☐ Yes ☐ No ☐ I don't know

27. Did you have an echocardiogram? ☐ Yes ☐ No ☐ I don't know

28. Did you have a multi-gated acquistion (MUGA) scan? ☐ Yes ☐ No ☐ I don't know

29. Was your LDL cholesterol greater than 130 at the time of diagnosis? ☐ Yes ☐ No ☐ I don't know

30. Is your LDL cholesterol greater than 130 now? ☐ Yes ☐ No ☐ I don't know

31. Was your HDL cholesterol less than 40 at the time of diagnosis? ☐ Yes ☐ No ☐ I don't know

32. Is your HDL cholesterol less than 40 now? ☐ Yes ☐ No ☐ I don't know

33. Were you on cholesterol medications at the time of diagnosis? ☐ Yes ☐ No ☐ I don't know

STUDY ID: ☐☐☐☐ PATIENT ID: ☐☐☐☐☐☐

34. Have you had cardiac surgery? ☐ Yes ☐ No
   34a. If yes:
   What type? ________________ Date ☐☐ / ☐☐ / ☐☐☐☐ (mm dd yyyy)

35. Have you had cardiac catheterization? ☐ Yes ☐ No
   35a. If yes:
   Was it: ☐ Normal ☐ Abnormal Date ☐☐ / ☐☐ / ☐☐☐☐ (mm dd yyyy)

36. Are you on cholesterol medications now? ☐ Yes ☐ No ☐ I don't know

37. Was your blood pressure consistently greater than 140/90 at the time of diagnosis? ☐ Yes ☐ No ☐ I don't know

38. Is your blood pressure consistently greater than 140/90 now? ☐ Yes ☐ No ☐ I don't know

39. Were you on blood pressure medications at the time of diagnosis? ☐ Yes ☐ No ☐ I don't know

40. Are you on blood pressure medications now? ☐ Yes ☐ No ☐ I don't know

41. Were you ever diagnosed with diabetes? ☐ Yes ☐ No ☐ I don't know
   If yes,
   Was it Type 1? ☐ Yes ☐ No
   Was it Type 2? ☐ Yes ☐ No
   Was it Gestational? ☐ Yes ☐ No

42. Have you ever been diagnosed with.......
   a. high blood pressure? ☐ Yes ☐ No ☐ I don't know
   b. a heart attack? ☐ Yes ☐ No ☐ I don't know
   c. a heart failure? ☐ Yes ☐ No ☐ I don't know
   d. high cholesterol? ☐ Yes ☐ No ☐ I don't know

43 Has your father ever been diagnosed with a heart attack? ☐ Yes ☐ No ☐ I don't know
   a. Was he diagnosed before the age of 65? ☐ Yes ☐ No ☐ I don't know

44. Has your mother ever been diagnosed with a heart attack? ☐ Yes ☐ No ☐ I don't know
   a. Was she diagnosed before the age of 65? ☐ Yes ☐ No ☐ I don't know

45. Have any of your grandparents ever been diagnosed with a heart attack? ☐ Yes ☐ No ☐ I don't know
   a. Were any of your grandmothers diagnosed before the age of 65? ☐ Yes ☐ No ☐ I don't know
   b. Were any of your grandfathers diagnosed before the age of 55? ☐ Yes ☐ No ☐ I don't know

46. Has anyone else been diagnosed with a heart attack? ☐ Yes ☐ No
   46a. If yes, who? ☐☐☐☐☐☐☐☐☐☐☐☐☐☐☐☐☐☐

20364

UNIVERSITY OF SOUTH FLORIDA

STUDY ID: ☐☐☐☐ PATIENT ID: ☐☐☐☐☐☐

Treatment

47. Were you initially treated with chemotherapy? ☐ Yes ☐ No ☐ I don't know

47a. Do you know what chemotherapy was used?

Did you receive.............	Date (mm / dd / yyyy)	How many cycles
☐ Adriamycin/doxorubicin	__ __ / __ __ / __ __ __ __	__ __
☐ Cytoxan	__ __ / __ __ / __ __ __ __	__ __
☐ AC	__ __ / __ __ / __ __ __ __	__ __
☐ Taxol/Paclitaxel	__ __ / __ __ / __ __ __ __	__ __
☐ Taxotere/docetaxel	__ __ / __ __ / __ __ __ __	__ __
☐ 5-Fu/Fluorouracil	__ __ / __ __ / __ __ __ __	__ __
☐ Epirubicin	__ __ / __ __ / __ __ __ __	__ __
☐ FEC	__ __ / __ __ / __ __ __ __	__ __
☐ Methotrexate	__ __ / __ __ / __ __ __ __	__ __
☐ CMF	__ __ / __ __ / __ __ __ __	__ __
☐ Other ____________	__ __ / __ __ / __ __ __ __	__ __

48. Did you receive hormonal therapy? ☐ Yes ☐ No ☐ I don't know

48a. Do you know which hormonal therapy was used?

Did you receive.............	Date (mm / dd / yyyy)	How many years
☐ Tamoxifen	__ __ / __ __ / __ __ __ __	__ __
☐ Arimidex (Anastrozole)	__ __ / __ __ / __ __ __ __	__ __
☐ Femara (Letrozole)	__ __ / __ __ / __ __ __ __	__ __
☐ Faslodex	__ __ / __ __ / __ __ __ __	__ __
☐ Aromasin (Exemestane)	__ __ / __ __ / __ __ __ __	__ __
☐ Other ____________	__ __ / __ __ / __ __ __ __	__ __

STUDY ID: ☐☐☐☐ PATIENT ID: ☐☐☐☐☐☐

49. Did you receive radiation? ☐ Yes ☐ No ☐ I don't know

If yes:

49a. Was it only to the primary tumor? ☐ Yes ☐ No ☐ I don't know

If **YES**, when? ☐☐ / ☐☐ / ☐☐☐☐ (mm / dd / yyyy)

Length of Treatment ☐☐ years

49b. Was it to the bone? ☐ Yes ☐ No ☐ I don't know

If **YES**, when? ☐☐ / ☐☐ / ☐☐☐☐ (mm / dd / yyyy)

Length of Treatment ☐☐ years

49c. Was it to another organ? ☐ Yes ☐ No ☐ I don't know

______________________ **(please specify)**

If **YES**, when? ☐☐ / ☐☐ / ☐☐☐☐ (mm / dd / yyyy)

Length of Treatment ☐☐ years

50. Did you receive surgery? ☐ Yes ☐ No ☐ I don't know

If yes:

50a. Was it a lumpectomy? ☐ Yes ☐ No ☐ I don't know

If **YES**, when? ☐☐ / ☐☐ / ☐☐☐☐ (mm / dd / yyyy)

50b. Was it mastectomy? ☐ Yes ☐ No ☐ I don't know

If **YES**, when? ☐☐ / ☐☐ / ☐☐☐☐ (mm / dd / yyyy)

50c. Was it another type? ______________________ ☐ Yes ☐ No ☐ I don't know

(please specify)

If **YES**, when? ☐☐ / ☐☐ / ☐☐☐☐ (mm / dd / yyyy)

50d. Did you have an excisional biopsy? ☐ Yes ☐ No ☐ I don't know

If **YES**, when? ☐☐ / ☐☐ / ☐☐☐☐ (mm / dd / yyyy)

50e. Did you have a sentinal lymph node biopsy? ☐ Yes ☐ No ☐ I don't know

If **YES**, when? ☐☐ / ☐☐ / ☐☐☐☐ (mm / dd / yyyy)

STUDY ID: ☐☐☐☐ PATIENT ID: ☐☐☐☐☐☐

50f. Did you have a lymph node dissection? ☐ Yes ☐ No ☐ I don't know If **YES**, when? ☐☐ / ☐☐ / ☐☐☐☐ (mm / dd / yyyy)

If so, how many lymph nodes were removed? ☐☐

How many lymph nodes were involved with cancer? ☐☐

Metastatic Disease

51. Did you have recurrence or metastasis of your cancer (same type as original)? ☐ Yes ☐ No ☐ I don't know

51a.Was it in your bone? ☐ Yes ☐ No ☐ I don't know

If **YES**, when? ☐☐ / ☐☐ / ☐☐☐☐ (mm / dd / yyyy)

51b. Was it in your liver? ☐ Yes ☐ No ☐ I don't know

If **YES**, when? ☐☐ / ☐☐ / ☐☐☐☐ (mm / dd / yyyy)

51c. Was it somewhere else? ☐ Yes ☐ No ☐ I don't know

______________________ **(please specify)**

If **YES**, when? ☐☐ / ☐☐ / ☐☐☐☐ (mm / dd / yyyy)

52. Did you develop a second cancer? ☐ Yes ☐ No ☐ I don't know

52a. Was it breast? ☐ Yes ☐ No ☐ I don't know

If **YES**, when? ☐☐ / ☐☐ / ☐☐☐☐ (mm / dd / yyyy)

52b. Was it lobular? ☐ Yes ☐ No ☐ I don't know

If **YES**, when? ☐☐ / ☐☐ / ☐☐☐☐ (mm / dd / yyyy)

52c. Was it ductal? ☐ Yes ☐ No ☐ I don't know

If **YES**, when? ☐☐ / ☐☐ / ☐☐☐☐ (mm / dd / yyyy)

52d. Was it another type? ☐ Yes ☐ No ☐ I don't know

______________________ **(please specify)**

If **YES**, when? ☐☐ / ☐☐ / ☐☐☐☐ (mm / dd / yyyy)

USF UNIVERSITY OF SOUTH FLORIDA

STUDY ID: ☐☐☐☐ PATIENT ID: ☐☐☐☐☐☐

52e. Was it ovarian? **(Females only)** ☐ Yes ☐ No ☐ I don't know

If **YES**, when? ☐☐ / ☐☐ / ☐☐☐☐ (mm / dd / yyyy)

52f. Was it colon? ☐ Yes ☐ No ☐ I don't know

If **YES**, when? ☐☐ / ☐☐ / ☐☐☐☐ (mm / dd / yyyy)

52g. Was it another type? ☐ Yes ☐ No ☐ I don't know

________________________ **(please specify)**

If **YES**, when? ☐☐ / ☐☐ / ☐☐☐☐ (mm / dd / yyyy)

Treatment of metastatic or new malignancy

53. Are you currently being treated for cancer? ☐ Yes ☐ No

If yes, when did you start? ☐☐ / ☐☐☐☐ (mm / yyyy)

54. Are you being treated with chemotherapy? ☐ Yes ☐ No

If yes, when did you start? ☐☐ / ☐☐☐☐ (mm / yyyy)

55. Are you being treated with hormonal therapy? ☐ Yes ☐ No

If yes, when did you start? ☐☐ / ☐☐☐☐ (mm / yyyy)

56. Do you know what chemotherapy is being used? ________________________________

Length of treatment

57. Are you receiving radiation? ☐ Yes ☐ No ☐ I don't know ☐☐

If **YES**, when did it start? ☐☐ / ☐☐ / ☐☐☐☐ (mm / dd / yyyy)

57a. Is it only to the primary tumor? ☐ Yes ☐ No ☐ I don't know ☐☐

If **YES**, when did it start? ☐☐ / ☐☐ / ☐☐☐☐ (mm / dd / yyyy)

57b. Is it to bone? ☐ Yes ☐ No ☐ I don't know ☐☐

If **YES**, when did it start? ☐☐ / ☐☐ / ☐☐☐☐ (mm / dd / yyyy)

57c. Is it to another organ? ☐ Yes ☐ No ☐ I don't know ☐☐

________________________ **(please specify)**

If **YES**, when did it start? ☐☐ / ☐☐ / ☐☐☐☐ (mm / dd / yyyy)

UNIVERSITY OF SOUTH FLORIDA

20364

STUDY ID: [ ][ ][ ][ ] PATIENT ID: [ ][ ][ ][ ][ ][ ]

Thank you for completing the questionnaire. Please provide the following contact information so we can reach you for further information if necessary. Please note that if you decide to complete this section, our study will not identify you by name but by study number. All of your information will be kept confidential.

Name: ____________________

Phone Number: [ ][ ][ ] - [ ][ ][ ] - [ ][ ][ ][ ]

Email address: ____________________

Best time to be contacted: Early Morning Late Morning Afternoon Evening Night time
(Please circle one)
